# Dietary quality and dietary greenhouse gas emissions in the USA: a comparison of the planetary health diet index, healthy eating index-2015, and dietary approaches to stop hypertension

**DOI:** 10.1186/s12966-024-01581-y

**Published:** 2024-04-02

**Authors:** Sarah M. Frank, Lindsay M Jaacks, Katie Meyer, Donald Rose, Linda S Adair, Christy L Avery, Lindsey Smith Taillie

**Affiliations:** 1 Global Academy of Agriculture and Food Systems, Royal (DICK) School of Veterinary Studies, The University of Edinburgh, Edinburgh, Scotland; 2 Carolina Population Center, University of North Carolina at Chapel Hill, 123 W Franklin St, Room 2107, Chapel Hill, NC 27514 USA; 3 Department of Nutrition, Gillings School of Global Public Health, University of North Carolina at Chapel Hill, Chapel Hill, NC USA; 4 Nutrition Research Institute, University of North Carolina at Chapel Hill, Chapel Hill, NC USA; 5 Tulane Nutrition, School of Public Health and Tropical Medicine, Tulane University, New Orleans, LA USA; 6 Department of Epidemiology, Gillings School of Global Public Health, University of North Carolina at Chapel Hill, Chapel Hill, NC USA

**Keywords:** EAT-*Lancet* Commission, Dietary patterns, Planetary Health Diet, Healthy eating index, Dietary approaches to stop hypertension, Dietary greenhouse gas emissions, NHANES, 24-hour recalls

## Abstract

**Background:**

The Planetary Health Diet Index (PHDI) measures adherence to the dietary pattern presented by the EAT-*Lancet* Commission, which aligns health and sustainability targets. There is a need to understand how PHDI scores correlate with dietary greenhouse gas emissions (GHGE) and how this differs from the carbon footprints of scores on established dietary recommendations. The objectives of this study were to compare how the PHDI, Healthy Eating Index-2015 (HEI-2015) and Dietary Approaches to Stop Hypertension (DASH) relate to (a) dietary GHGE and (b) to examine the influence of PHDI food components on dietary GHGE.

**Methods:**

We used life cycle assessment data from the Database of Food Recall Impacts on the Environment for Nutrition and Dietary Studies to calculate the mean dietary GHGE of 8,128 adult participants in the 2015–2016 and 2017–2018 cycles of the National Health and Nutrition Examination Survey (NHANES). Poisson regression was used to estimate the association of (a) quintiles of diet score and (b) standardized dietary index Z-scores with dietary GHGE for PHDI, HEI-2015, and DASH scores. In secondary analyses, we used Poisson regression to assess the influence of individual PHDI component scores on dietary GHGE.

**Results:**

We found that higher dietary quality on all three indices was correlated with lower dietary GHGE. The magnitude of the dietary quality-dietary GHGE relationship was larger for PHDI [-0.4, 95% CI (-0.5, -0.3) kg CO_2_ equivalents per one standard deviation change] and for DASH [-0.5, (-0.4, -0.6) kg CO_2_-equivalents] than for HEI-2015 [-0.2, (-0.2, -0.3) kg CO_2_-equivalents]. When examining PHDI component scores, we found that diet-related GHGE were driven largely by red and processed meat intake.

**Conclusions:**

Improved dietary quality has the potential to lower the emissions impacts of US diets. Future efforts to promote healthy, sustainable diets could apply the recommendations of the established DASH guidelines as well as the new guidance provided by the PHDI to increase their environmental benefits.

**Supplementary Information:**

The online version contains supplementary material available at 10.1186/s12966-024-01581-y.

## Background

Over the past 50 years, global dietary shifts and accompanying changes in food production have played a significant role in environmental degradation [1, 2]. Specifically, increased consumption of ruminant meats has led to increased diet-related greenhouse gas emissions (GHGE) [3, 4], as has increasing intake of ultra-processed foods high in refined starches, added sugars, fats and oils that provide little nutritional value to the diet but demand significant environmental resources that could otherwise be used for healthy food production [2, 5]. Food production makes up 16% of all GHGE in the US, a country for which per-capita emissions are more than three times the global average [6, 7]. Beyond environmental impact, these dietary shifts are also a key part of the nutrition transition and are a major contributor to the burden of non-communicable disease in the US [8, 9].

In this context, dietary guidelines are one strategy to alter consumer behavior and the composition of federal food policies, with the potential for food production to shift in response to changing dietary preferences [10]. One such set of dietary recommendations is those released by the EAT-*Lancet* Commission on Food, Planet, Health, which are unique in their explicit aim to jointly promote sustainability and human health. Their recommendations are an omnivorous diet that consists mostly of plant-based foods and allows for small amount of animal products [1]. The Planetary Health Diet Index (PHDI) is a dietary quality tool designed to measure adherence to these recommendations [11].

Previous research in the US has consistently found that healthy plant-based diets are associated with lower diet-related GHGE, while unhealthy plant-based diets do not necessarily have the same environmental benefits [12–14]. In this context, the PHDI can fill an important gap, as it is a novel *a priori* measure that considers both environmental sustainability and health outcomes. But to date, no study has examined how American diets align with the recommendations of the PHDI and how PHDI scores in the US correlate with dietary GHGE. Other commonly used dietary indices in the US primarily exist to inform health outcomes. For example, the Healthy Eating Index (HEI) quantifies adherence to the Dietary Guidelines for Americans (DGAs), which are designed to promote nutrient adequacy and prevent disease in the American population [15]. Results on the associations between diet quality as measured by the DGAs and diet-related GHGE have been mixed [13, 16–18]. Another example is the Dietary Approaches to Stop Hypertension (DASH), which is designed to prevent cardiovascular disease [19]. To justify incorporating more climate-focused dietary recommendations into US food policies—such as those recommended by the EAT-*Lancet* Commission and captured by the PHDI—there is a need to assess how current dietary patterns in the US align with this guidance and compare how diet-related GHGEs of individuals adhering to PHDI differ from adherence to dietary recommendations already in use.

Additionally, certain foods—such as red meat—are known to have much higher emissions impacts than others [4]. Therefore, it is important to understand whether the associations with indices such as PHDI and dietary GHGE are driven by overall differences in dietary quality, or if individual components are driving these differences.

The primary objective of this study was to compare the performance of PHDI with the Healthy Eating Index-2015 (HEI-2015) and Dietary Approaches to Stop Hypertension (DASH) with respect to diet-related GHGE. We further examine the influence of individual PHDI food components on diet-related GHGE.

## Methods

### Study population

The US National Health and Nutrition Examination Survey (NHANES) is a repeated cross-sectional survey that obtains a nationally-representative sample of the civilian, non-institutionalized population of the United States [20]. Two cycles of NHANES are recommended to obtain reliable estimates of population-level means [21, 22], so we included data from the two most recently available NHANES cycles unaffected by the COVID-19 pandemic. Eligible participants were non-pregnant or lactating individuals aged 20 years or older who participated in the 2015–2016 or 2017–2018 NHANES cycle and for whom two days of valid dietary intake data were available. Participants whose mean total energy intake was < 500 kcal or > 8000 kcal/day were excluded [23].

### Assessment of dietary intake

Trained NCHS interviewers used the Automated Multiple Pass Method to gather 24-hour dietary recall data on all foods and beverages consumed by participants on the previous day [24]. The second dietary interview was conducted via an unannounced phone call three to ten days after the initial face-to-face interview.

Twenty-four hour dietary recall data were merged to the Food Patterns Equivalent Database (FPED), which uses the USDA Food Composition Table to categorize foods into the 37 USDA Food Pattern Components. For single-ingredient food items, FPED assigns foods directly to the corresponding component. For multi-ingredient foods with ingredients from more than one component, FPED disaggregates these items into their component ingredients using standard recipe files [25]. We converted the food pattern equivalents measurements (e.g., cups, ounces, teaspoons) to grams via a method described elsewhere [26]. Because cow’s milk is approximately 90% water, we used FPED’s cup-equivalents of dairy rather than grams of dairy to better represent the nutrient density and environmental impact of the various dairy foods (e.g., milk vs. cheese) [14].

Dietary recall data also provided estimates of participants’ total energy intake.

### Dietary indices

To validate the PHDI’s recommendations with respect to emissions, we compared the PHDI with two other commonly used dietary indices, the Healthy Eating Index-2015 (HEI-2015) [15] and the Dietary Approaches to Stop Hypertension (DASH) [19].

The PHDI measures adherence to the recommendations of the EAT-*Lancet* Commission Scientific Report [1]. Its purpose is to provide evidence-based recommendations that promote human health and operate within planetary boundaries. PHDI consists of fourteen components scored from 0 to 10 points each, six of which are adequacy components and eight of which are moderation components. Therefore, the theoretical range of the PHDI is 0 to 140. Additional details on the derivation of the PHDI are available elsewhere [11, 26].

The HEI-2015 is a quantitative measure of adherence to the US Dietary Guidelines for Americans (DGAs), which are dietary recommendations published by the federal government to help the American population meet nutritional requirements, prevent chronic disease, and promote health. They are used as the basis for federal food and nutrition policy [15, 27]. HEI-2015 consists of thirteen components, nine of which are adequacy components (three scored from 0 to 10 points, six scored from 0 to 5 points) and four of which are moderation components (all scored from 0 to 10 points). The theoretical range of the HEI-2015 is 0-100 points, and the minimum and maximum scoring criteria for each food group are described in detail elsewhere [28, 29].

For both PHDI and HEI-2015, the scoring for components is assigned based on *a priori* quantities and participant intakes between the minimum and maximum were scored proportionately.

DASH is specifically designed to maintain a healthy blood pressure and has been adapted in settings throughout the globe. It consists of eight components, five of which are adequacy components and three of which are moderation components. Scores for each category range from 1 to 5, as described by Fung et al. [30]. Unlike PHDI or HEI-2015, DASH scores are defined by the underlying distribution of component intake in the study population, rather than by *a priori* quantities. DASH values range from 8 to 40 [29, 31].

For all three indices, component scores were summed to create a total index value, with a higher value indicating better adherence to the dietary recommendations and higher dietary quality. A summary table of the components of the three indices is available in Supplemental Table 1.

### Calculation of diet-related GHGE

We used data from the database of Food Recall Impacts on the Environment for Nutrition and Dietary Studies (*dataFRIENDS*) to obtain diet-related GHGE estimates. The methodology of *dataFRIENDS* is described in detail elsewhere [32]. Briefly, *dataFRIENDS* relies on the linkage between the Food Commodities Intake Database (FCID) with individual dietary data from NHANES. FCID was a database developed by the US Environmental Protection Agency to enable a diet-level analysis of food commodities. The FCID database contains a recipe file that links foods reported in NHANES to ingredients in the form of 332 commodities. To create *dataFRIENDS*, Heller and colleagues conducted a literature review and calculated the greenhouse gas emissions associated with each of the 332 commodities in FCID from seed to farm gate [17, 32]. Additionally, proxy assignments were made when direct matches were unavailable, and adjustments were made to account for differences in mass basis (for example, excluding inedible portions) [17, 32]. A team of three trained research assistants, led by a member of the original dataFRIENDS (version 1.0) team, applied the same methodology used to derive the publicly-available version of dataFRIENDS to obtain estimates for the 2015–2016 (v2.0) [33] and 2017–2018 (v3.0) cycles of NHANES.

GHGE data are reported in kilogram CO_2_-equivalents (CO_2_-eq) per edible kilogram (kg) of food, which reflect global warming potential. Using the *dataFRIENDS* linkage described above, the total GHG can be calculated for each NHANES participant by summing over all food items reported in the 24-hour recalls. Because we used the mean of two days of dietary recall to derive the dietary index exposure variable, we calculated mean emissions from two days of dietary recall as the outcome.

### Sociodemographic characteristics

All sociodemographic information was self-reported as part of a standardized questionnaire. Age data were modeled in continuous years. Income data were classified using the Poverty Income Ratio (PIR), a measure of family income relative to the Federal Poverty Level that accounts for household size. Income was categorized as PIR 0–185%, PIR 186–399%, PIR ≥ 400%, and Missing (due to high missingness in self-reported income, 8.1%) [34]. Education was reported in continuous years and classified as high school equivalent or lower, some college, and college degree or higher [35]. Race/ethnicity data were self-reported via categorical selection and classified as Non-Hispanic white, Non-Hispanic Black, Hispanic, and Other race/ethnicity (including Multiracial) [34, 36].

### Analyses

In descriptive analyses, participants were classified into quintile of dietary GHGE and we compared the scores for each component in the highest vs. lowest quintile of GHGE.

Participants were classified into quintiles for each diet index (PHDI, HEI-2015, and DASH) and for dietary GHGE. Because of the skew in the diet-related GHGE outcome, Poisson regression models [37] were used to estimate the association between quintile of diet score and dietary GHGE. To directly compare the dietary indices, we created a standardized variable (Z-score) for each index and included this variable as a continuous exposure in Poisson regression. We compared the results from the continuous analysis using adjusted Wald tests. All models were adjusted for total energy intake.

To assess the influence of individual PHDI components on dietary GHGE, we conducted exploratory data analysis in which we regressed continuous dietary GHGE on each component score, both alone and controlling for overall PHDI score. To assess whether any individual PHDI components drove the observed associations of PHDI with dietary GHGE, we created 14 modified versions of the PHDI that was total PHDI score minus each respective component [38]. We then repeated our main analyses and regressed continuous GHGE on each of these modified scores. As in the main analyses, in these exploratory analyses all models were adjusted for total energy intake.

All analyses were conducted in Stata 17.0. We used a statistical significance level of *p* < 0.05 and applied the Bonferroni correction for multiple testing where appropriate. We accounted for the multistage sampling design of NHANES using the primary sampling unit and stratum variables and applied complex survey weights.

## Results

The final sample included 8,128 eligible participants (Table 1, Supplemental Fig. 1). The range of PHDI values was 18–125 on a scale from 0 to 140, whereas HEI-2015 values ranged from 15 to 99 on a scale of 0-100, and DASH spanned the theoretical range of 8 to 40.

**Fig. 1:**
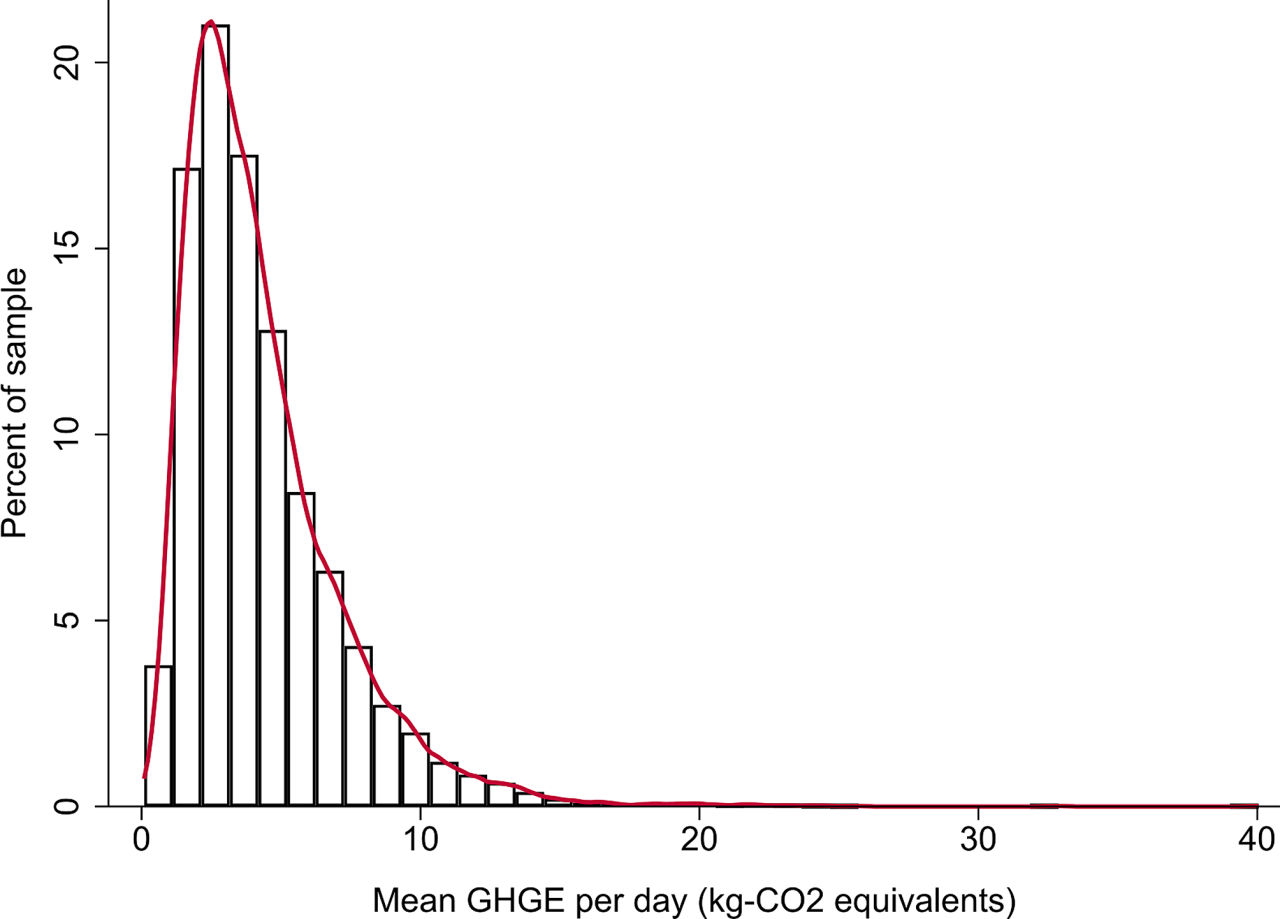
Histogram of mean daily greenhouse gas emissions (GHGE) in kilograms-CO_2_ equivalents, National Health and Nutrition Examination Survey 2015–2018

The distribution of per-capita dietary GHGE was skewed right, and median GHGE was 3.8 (IQR: 2.5–5.7) kg CO_2_-equivalents per person per day (Fig. 1).

**Table 1:** Characteristics of eligible participants with two days of dietary recall data, National Health and Nutrition Examination Survey 2015–2018^*^

**Sex**	
Male	49.1 (3954)
Female	50.9 (4174)
**Mean age (SD)**, years	48.6 (15.6)
**Educational attainment**	
High school equivalent or lower	35.5 (3425)
Some college	32.1 (2575)
College degree or greater	32.4 (2121)
**Income**	
Poverty-to-Income Ratio < 185%	28.6 (3212)
Poverty-to-Income Ratio 185–399%	28.3 (2217)
Poverty-to-Income Ratio ≥ 400%	35.0 (1874)
Missing income information	8.1 (825)
**Race/ethnicity**	
Non-Hispanic white	64.1 (2949)
Non-Hispanic Black	11.1 (1873)
Hispanic	14.8 (2054)
Asian, Multiracial, and Other Non-Hispanic race/ethnicities	10.0 (1252)
**Median (IQR) Planetary Health Diet Index score**	68 (59, 77)
**Median (IQR) Healthy Eating Index-2015 score**	53 (44, 63)
**Median (IQR) Dietary Approaches to Stop Hypertension score**	24 (19, 28)
**Median (IQR) diet-related greenhouse gas emissions**	3.9 (2.6, 5.8)

^*^ Values are weighted % (unweighted N) unless otherwise noted. Weighted % accounts for complex survey weights

We observed several differences in emissions by sociodemographic characteristics (Table 2). Mean dietary GHGE were higher for men than for women and for people with the highest levels of income, relative to those with the lowest income. Black individuals had lower dietary GHGE than any other racial/ethnic group. We did not observe any differences in diet-related GHGE by age or educational attainment.

**Table 2:** Mean diet-related greenhouse gas emissions by sociodemographic characteristic, National Health and Nutrition Examination Survey 2015–2018^*^

	Estimated Emissions (kg CO_2_-eq person^− 1^ day^− 1^) (95% CI)	p^†^
Age		
20–29	4.3 (4.1, 4.5)	--
30–39	4.4 (4.2, 4.6)	0.49
40–49	4.5 (4.3, 4.6)	0.15
50–59	4.5 (4.3, 4.7)	0.07
60–69	4.5 (4.2, 4.8)	0.11
70–79	4.3 (4.1, 4.5)	0.98
80 or older	4.1 (3.9, 4.2)	0.10
Sex		
Male	4.7 (4.5, 4.9)	--
Female	4.1 (4.1, 4.2)	< 0.001
Income		
PIR≤1.30	4.3 (4.1, 4.4)	--
PIR > 1.30–3.50	4.4 (4.2, 4.6)	0.04
PIR > 3.50	4.5 (4.3, 4.6)	0.002
Missing	4.5 (4.3, 4.7)	0.04
Education		
High school equivalent or lower	4.6 (4.5, 4.8)	--
Some college	4.6 (4.4, 4.7)	0.60
College graduate or above	4.4 (4.3, 4.6)	0.06
Race/Ethnicity		
Non-Hispanic White	4.4 (4.3, 4.6)	--
Non-Hispanic Black	4.1 (4.0, 4.3)	< 0.001
Hispanic	4.5 (4.4, 4.6)	0.39
Other race/ethnicity (including Multiracial)	4.3 (4.1, 4.5)	0.15

^*^ Poisson regression models adjusted for total energy intake

^†^ p-value for the contrast with the reference category

**Fig. 2:**
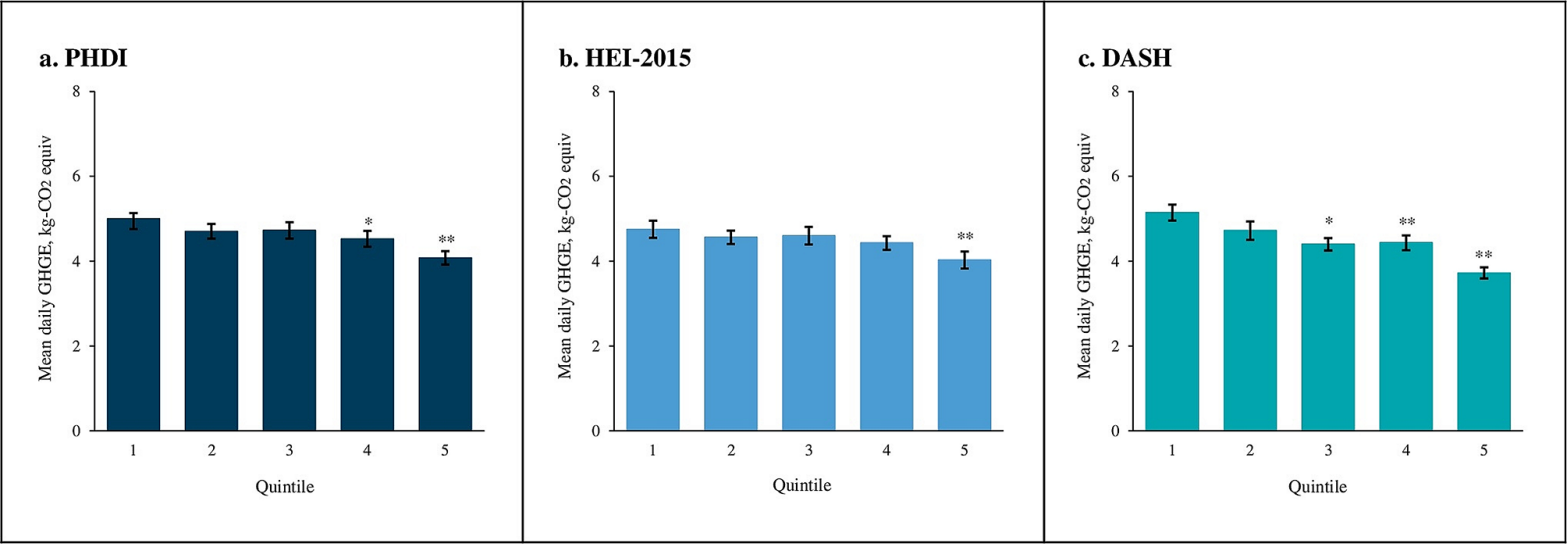
Predicted greenhouse gas emissions by quintile of Planetary Health Diet Index (PHDI), Healthy Eating Index-2015 (HEI-2015), and Dietary Approaches to Stop Hypertension (DASH), National Health and Nutrition Examination Survey 2015–2018^*^ ^*^ Poisson regression models adjusted for total energy intake ^†^ *p < 0.01, **p < 0.001 for the difference from Quintile 1. With the application of the Bonferroni correction statistical significance is defined as *p* < 0.0125 (0.05/4 comparisons = 0.0125)

Being in a higher quintile of diet quality was associated with lower dietary GHGE for PHDI [5.0 (4.8, 5.2) in quintile 1 vs. 4.1 (3.9, 4.2) in quintile 5, *p* < 0.001, p_trend_<0.001], HEI-2015 [4.8 (4.6, 5.0) in quintile 1 vs. 4.1 (3.9, 4.3) in quintile 5, *p* < 0.001, p_trend_<0.001], and DASH [5.2 (5.0, 5.4) in quintile 1 vs. 3.8 (3.7, 3.9) in quintile 5, *p* < 0.001, p_trend_<0.001] (Fig. 2, Supplemental Table 2). In analyses with standardized scores as the continuous exposure variable, a higher dietary score was similarly associated with lower dietary GHGE for all three indices. However, the magnitude of the association for standardized DASH [-0.51, (-0.60, -0.42) kg CO_2_-equivalents lower] was stronger than that observed for PHDI [-0.37 (-0.45, 0.28) kg CO_2_-equivalents lower, *p* < 0.001]. Both DASH and PHDI had a larger inverse association with diet-related GHGE than HEI-2015 [-0.25, (-0.33, -0.16) kg CO_2_-equivalents lower, all *p* < 0.001].

Several PHDI components were also associated with GHGE. For the adequacy components whose intake is encouraged, higher non-starchy vegetables score was associated with higher diet-related GHGE [0.05 (95% CI: 0.02, 0.08) kg CO_2_-equivalents increase per one-unit increase in non-starchy vegetable score], while higher scores for all other adequacy components were associated with lower diet-related GHGE (Table 3). For the moderation components for which limited intakes are recommended, higher scores for poultry [0.07 (0.05, 0.08) kg CO_2_ equivalents], for saturated oils and *trans* fats [0.05 (0.03, 0.07) kg CO_2_ equivalents] and for added sugar and fruit juice [0.09 (0.07, 0.11) kg CO_2_ equivalents] were associated with an increase in diet-related GHGE. Higher scores for red and processed meat [-0.43 (-0.46, -0.40) kg CO_2_ equivalents] and for dairy [-0.04 (-0.07, -0.01) kg CO_2_ equivalents] were associated with lower diet-related GHGE (Table 3). Because of the inverse relationship between intake and dietary score for the moderation components, this means that a lower intake of poultry, saturated oils and *trans* fats and of added sugar and fruit juice is correlated with higher GHGE, and lower intake of red and processed meat and of dairy is associated with lower GHGE. The magnitude and direction of the results were robust when including overall PHDI score as an additional predictor variable in the models. When the component score for red and processed meat was subtracted from the calculation of PHDI score, the association between PHDI score and mean GHGE was no longer statistically significant.

**Table 3:** Association of Planetary Health Diet Index (PHDI) components with dietary greenhouse gas emissions (GHGE), National Health and Nutrition Examination Survey 2015–2018^*†^

	Component alone	Component controlling for total PHDI	PHDI score - component
*Adequacy components*			
Whole grains	-0.05^***^ (-0.06, -0.03)	-0.01 (-0.03, 0.01)	-0.03^***^ (-0.03, -0.02)
Whole fruits	-0.04^**^ (-0.06, -0.01)	0.01 (-0.01, 0.03)	-0.03^***^ (-0.03, -0.02)
Non-starchy vegetables	0.05^**^ (0.02, 0.08)	0.13^***^ (0.09, 0.16)	-0.04^***^ (-0.05, -0.03)
Nuts and seeds	-0.08^***^ (-0.11, -0.06)	-0.05^***^ (-0.07, -0.02)	-0.02^***^ (-0.03, -0.02)
Total legumes	-0.08^***^ (-0.12, -0.04)	-0.02 (-0.06, 0.01)	-0.03^**^ (-0.03, -0.02)
Unsaturated oils	-0.08^***^ (-0.11, -0.06)	-0.06^**^ (-0.09, -0.02)	-0.02^***^ (-0.03, -0.02)
*Moderation components*			
Starchy vegetables	-0.02 (-0.04, 0.01)	-0.01 (-0.04, 0.01)	-0.03^**^ (-0.03, -0.02)
Dairy	-0.04^*^ (-0.07, -0.01)	-0.03^*^ (-0.06. -0.01)	-0.03^***^ (-0.03, -0.02)
Red and processed meat	-0.43^***^ (-0.46, -0.40)	-0.42^***^ (-0.45, -0.39)	0.00 (-0.01, 0.01)
Poultry	0.07^***^ (0.05, 0.08)	0.09^***^ (0.07, 0.10)	-0.03^***^ (-0.04, -0.03)
Eggs	-0.02 (-0.05, 0.00)	0.00 (-0.03, 0.02)	-0.03^***^ (-0.03, -0.02)
Fish	-0.04 (-0.07, 0.01)	-0.02 (-0.05, 0.00)	-0.03^***^ (-0.03, -0.02)
Saturated oils and trans fats	0.05^***^ (0.03, 0.07)	0.10^***^ (0.08, 0.13)	-0.04^***^ (-0.04, -0.03)
Added sugar and fruit juice	0.09^***^ (0.07, 0.11)	0.15^***^ (0.13, 0.18)	-0.04^***^ (-0.05, -0.03)

^*^ Values are coefficients from energy-adjusted Poisson regression and represent the predicted change in GHGE per a one-point increase in component or in modified PHDI score

^†^ * *p* < 0.05, ** *p* < 0.01, *** *p* < 0.001 for the difference from 0

## Discussion

We found that, in this nationally-representative survey of adults living in the US, improved dietary quality across the three dietary indices we examined was associated with lower GHGE. The inverse relationship between dietary quality and lower diet-related GHGE is consistent with several recent studies published in the US [12, 17]. Another NHANES study found that vegan, vegetarian, and pescetarian diets had higher dietary quality and lower diet-related GHGE than omnivorous, keto, or paleo diets [13]. Additionally, among participants who consumed omnivorous diets, an increase in DASH-score was associated with a decrease in diet-related GHGE [13]. Results from the Nurses’ Health Study similarly found that higher diet quality measured by the Alternative Healthy Eating Index and the Plant-based Diet Index were associated with lower diet-related GHGE [14]. Outside the US, diet quality has similarly been associated with lower GHGE in other high-income settings [39, 40].

However, not all studies have found that diets lower in GHGE necessarily have higher dietary quality [16, 41, 42]. The components analysis in our study reflects the inherent complexity of diet and the nuanced ways in which components influence estimates of nutritional quality and diet-related GHGE. Similar to our results, other studies have found that healthy plant-based foods such as whole grains, fruits, nuts, and legumes are associated with lower diet-related GHGE [17, 39, 41], but so are added sugars [17, 41] and ultra-processed foods [43–45]. At the same time, non-starchy vegetable intake has been associated with higher diet-related GHGE in other studies as well [17]. As outlined in the EAT-*Lancet* Commission report, policy actions to improve the environmental sustainability diet therefore cannot target only diet-related GHGE, but must consider trade-offs with nutrition as well [1].

However, one component—red and processed meat—had a much larger impact on diet-related GHGE than any other PHDI component. Red and processed meat have high production-associated GHGE [4], and diets high in this component are consistently found to have higher diet-related GHGE [46, 47]. The high emissions of red and processed meat could also explain why the associations of GHGE with PHDI and for DASH were stronger than those with HEI: PHDI and DASH both consider red meat as a moderation component to limit, whereas HEI does not. While red meat is a source of nutrients such iron and vitamin B12, at high intakes such as those observed in the US, it is also correlated with cardiovascular disease [48], type II diabetes [49], and certain cancers [50]. Moreover, in the US and other high-income contexts with high intake of animal-sourced foods, substituting red and processed meat in favor of more plant-based foods is estimated to have benefits for nutrient adequacy [51]. For the US context, dietary guidelines that recommend limited intake red and processed meat could reduce diet-related GHGE and improve population health.

The present study has several limitations. First, the life cycle assessment data are only for cradle-to-farm gate for the vast majority of foods [17, 32]. They generally do not include GHGE associated with packaging or transportation and are therefore an underestimate of the total GHGE footprint of the included foods. Additionally, we use two days 24-hour dietary recall data, which does not capture usual intake for individuals. However, the use of NHANES survey weights allow us to obtain nationally-representative, population-level estimates for the three dietary quality indices and for the PHDI component scores. Furthermore, we did not do a components-level analysis for HEI-2015 or for DASH. However, the focus of this manuscript is to validate the PHDI, and to see how its recommendations may be applied in a US context. We also do not examine how dietary patterns correlate other environmental indicators, such as water use, land use, or biodiversity loss. Future research should examine the relationship of these environmental indicators with dietary quality in the US.

## Conclusions

Better dietary quality is associated with lower diet-related GHGE, with stronger associations for both PHDI and DASH than for HEI-2015. Red and processed meat—which is a moderation component for both PHDI and DASH—had the strongest influence on dietary GHGE. Future efforts to promote healthy, sustainable diets should reframe red and processed meat as a moderation component and could look to the established DASH guidelines as well as the new guidance provided by the PHDI.

### Electronic supplementary material

Below is the link to the electronic supplementary material.

Supplementary Material 1

## Data Availability

The datasets generated and/or analysed during the current study are available in the NHANES repository [https://www.cdc.gov/nchs/nhanes/index.htm].

## Abbreviations

dataFRIENDS: database of Food Recall Impacts on the Environment for Nutrition and Dietary Studies
DASH: Dietary Approaches to Stop Hypertension
DGAs: Dietary Guidelines for Americans
FPED: Food Patterns Equivalents Database
GHGE: Greenhouse gas emissions
HEI: Healthy Eating Index
NHANES: National Health and Nutrition Examination Survey
PHDI: Planetary Health Diet Index
TEI: Total Energy Intake
USDA: US Department of Agriculture
