# Supplementary material for: Dietary quality and dietary greenhouse gas emissions in the USA: a comparison of the planetary health diet index, healthy eating index-2015, and dietary approaches to stop hypertension
Source: Int J Behav Nutr Phys Act. 2024 Apr 2;21:36. doi: 10.1186/s12966-024-01581-y (PMC10988877; doi:10.1186/s12966-024-01581-y)
Supplement: Supplementary file 1 — Supplementary Material 1 [file 12966_2024_1581_MOESM1_ESM.docx]

**Dietary Quality and Dietary Greenhouse Gas Emissions in the USA: A Comparison of the Planetary Health Diet Index, Healthy Eating Index-2015, and Dietary Approaches to Stop Hypertension**

Sarah M Frank, Lindsay M Jaacks, Linda S Adair, Christy L Avery, Katie Meyer, Donald Rose, Lindsey Smith Taillie

[**Supplemental Table 1:** Comparison of Planetary Health Diet Index (PHDI), Healthy Eating Index-2015 (HEI-2015) and Dietary Approaches to Stop Hypertension (DASH) dietary components 1](#_Toc138493062)

[**Supplemental Figure 1**: Flowchart of participant inclusion, National Health and Nutrition Examination Survey 2015-2018 2](#_Toc138493063)

[**Supplemental Table 2:** Predicted mean greenhouse gas emissions by quintile of Planetary Health Diet Index (PHDI), Healthy Eating Index-2015 (HEI-2015), and Dietary Approaches to Stop Hypertension (DASH), National Health and Nutrition Examination Survey 2015-2018^*^ 3](#_Toc138493064)

| Supplemental Table 1: Comparison of Planetary Health Diet Index (PHDI), Healthy Eating Index-2015 (HEI-2015) and Dietary Approaches to Stop Hypertension (DASH) dietary components | | | |
| --- | --- | --- | --- |
| **Dietary Components** | **PHDI** | **HEI-2015^*^** | **DASH** |
| **Encouraged components** | | | |
| Grains | Whole grains | Whole grains | Whole grains |
| Fruits | Whole fruit  *(excluding juice)* | Whole fruit *(excluding juice)*; total fruit *(including juice)* | Total fruit  *(including juice)* |
| Vegetables | Vegetables  *(excluding starchy)* | Total vegetables;  greens and beans | Total vegetables |
| Proteins | Nuts; non-soy legumes; soybean/soy foods | Total protein foods; seafood and plant proteins | Total nuts and legumes |
| Dairy |  | Total dairy | Low-fat dairy |
| Fats & oils | Added fat:  unsaturated oils | Fatty acids (PUFAs + MUFAS)/ SFAs |  |
| **Discouraged components** | | | |
| Grains |  | Refined grains |  |
| Vegetables | Starchy vegetables |  |  |
| Proteins | Red/processed meat; poultry; eggs; fish |  | Red/processed meat |
| Dairy | Total dairy |  |  |
| Fats & oils | Added fat: saturated  oils, *trans* fat | Saturated fats |  |
| Sugar | Added sugar  and fruit juice | Added sugars  (excludes fruit juice) | Sugar-sweetened beverages |
| Sodium |  | Sodium | Sodium |
| * HEI will be updated upon the release of the HEI-2020, expected 2023 | | | |

Supplemental Figure 1: Flowchart of participant inclusion,

National Health and Nutrition Examination Survey 2015-2018


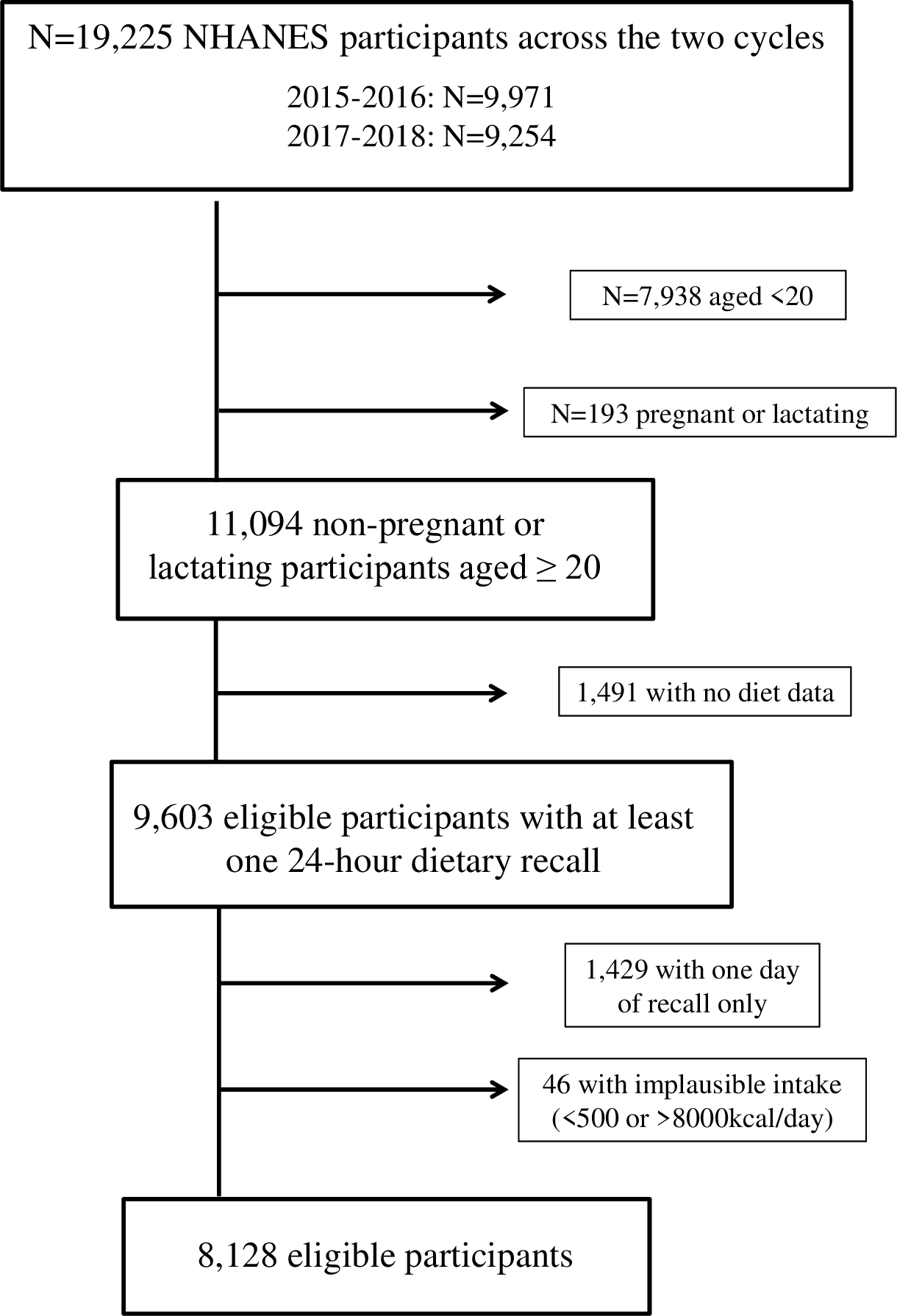


| Supplemental Table 2: Predicted mean greenhouse gas emissions by quintile of Planetary Health Diet Index (PHDI), Healthy Eating Index-2015 (HEI-2015), and Dietary Approaches to Stop Hypertension (DASH), National Health and Nutrition Examination Survey 2015-2018^*^ | | | | | | |
| --- | --- | --- | --- | --- | --- | --- |
|  | Quintile 1 | Quintile 2 | Quintile 3 | Quintile 4 | Quintile 5 | p for trend |
| PHDI | 5.0 (4.8, 5.2) | 4.7 (4.5, 4.9) | 4.7 (4.5, 4.9) | 4.5^*^ (4.3, 4.7) | 4.1^**^ (3.9, 4.2) | <0.001 |
| HEI-2015 | 4.8 (4.6, 5.0) | 4.6 (4.5, 4.8) | 4.7 (4.5, 4.9) | 4.5 (4.3, 4.7) | 4.1^**^ (3.9, 4.3) | <0.001 |
| DASH | 5.2 (5.0, 5.4) | 4.8 (4.6, 5.0) | 4.5^*^ (4.3, 4.6) | 4.5^*^ (4.3, 4.7) | 3.8^**^ (3.7, 3.9) | <0.001 |
| ^*^ Poisson regression models adjusted for total energy intake.  ^*^ ^†^ * p<0.01, ** p<0.001 for the difference from Quintile 1. With the application of the Bonferroni correction statistical significance is defined as p<0.0125 (0.05/4 comparisons = 0.0125) | | | | | | |
